# The Impact of Nerve Involvement on the Prognosis of Gastric Cancer Patients with Curative Gastrectomy: An International Multicenter Analysis

**DOI:** 10.1155/2021/8870562

**Published:** 2021-03-28

**Authors:** Kun Yang, Yu-Qing Dan, Yoon Young Choi, Zong-Guang Zhou, Woo Jin Hyung, Jian-Kun Hu, Sung Hoon Noh

**Affiliations:** ^1^Department of Gastrointestinal Surgery, West China Hospital, Sichuan University, China; ^2^Laboratory of Gastric Cancer, State Key Laboratory of Biotherapy/Collaborative Innovation Center of Biotherapy and Cancer Center, West China Hospital, Sichuan University, China; ^3^West China School of Medicine, Sichuan University, China; ^4^Department of Surgery, Severance Hospital, Yonsei University Health System, Yonsei University College of Medicine, Seoul, Republic of Korea; ^5^Department of Surgery, Gangnam Severance Hospital, Yonsei University Health System, Yonsei University College of Medicine, Seoul, Republic of Korea

## Abstract

**Background:**

Several studies have been conducted to investigate the association between the presence of perineural invasion (PNI) and overall survival (OS) of gastric cancer (GC) patients who underwent curative resection, but no consensus has been reached. This study is aimed at determining the prognostic significance of PNI in gastric cancer. *Study Design*. The data of 2969 patients with gastric cancer and who had undergone curative gastrectomy from 2006 to 2010 in two high-volume hospitals of China and Korea were retrospectively analyzed. PNI positivity was identified when carcinoma cells were found to infiltrate into the perineurium or neural fascicles. The relationships between PNI and other clinicopathological factors were evaluated, and survival analyses were performed.

**Results:**

The presence of PNI was detected in 1055 of the 2969 patients (35.5%). Nationality, age, tumor location, size of tumor, differentiation of the tumor, pT stage, pN stage, lymphatic invasion, and vascular invasion had been associated with PNI positivity. The mean survival time of patients with and without PNI was 62.5 months and 87.3 months, respectively (*P* < 0.001). However, the presence of PNI was not an independent prognostic factor for gastric cancer, except for patients in stage III (*P* = 0.037, hazard ratio: 1.21, 95% confidence interval: 1.01-1.44).

**Conclusion:**

PNI occurs frequently in patients with gastric cancer, and the incidence of PNI increases with the staging of the tumor. The presence of PNI can provide additional information in predicting the survival outcome for those with stage III tumors.

## 1. Introduction

Gastric cancer (GC) has been a great threat to public health. Although the incidence of GC has been gradually decreasing during the past few decades, it is still the fifth most common cancer and the third most lethal cancer worldwide [1]. There were more than 900,000 new cases diagnosed annually and more than 700,000 deaths caused by GC in a year, and the prognosis was not promising with the cumulative 5-year survival in most countries remaining in the narrow range of 25-30% in recent years—except for Japan and Korea [2].

Depth of invasion, lymph node metastasis, and distant metastasis were well acknowledged to be the most important prognostic risk factors. Despite the TNM staging system which has greatly helped the doctors to assess patients' prognosis and choose the stage-specific therapeutic strategy, the survival rates of patients with the same stage might have great differences, which means that other prognostic factors could impact the overall survival of GC patients besides the TNM stage [3, 4]. Moreover, some studies also reported similar survival curves of different TNM stages [5, 6]. Therefore, discovering potential new biological or pathological indicators in GC to provide a more precise prediction for patients' prognosis along with the existing prognostic factors would be necessary.

Perineural invasion (PNI) refers to the process by which cancer cells spread to the space surrounding a nerve. It is considered to be a prominent predictor for a more aggressive tumor phenotype and indicated poor prognosis in many carcinomas like prostatic cancer [7], bladder cancer [8, 9], and pancreatic cancer [10, 11]. Several studies have been conducted to identify the prognostic significance of PNI in GC, but the results are controversial [12–15]. The question of whether perineural invasion would provide additional prognostic information to the traditional TNM parameters is still debatable.

In this study, we investigated the relationships between PNI and other clinicopathological factors in GC and also assessed the prognostic value of PNI in GC, aiming to provide additional effective prognostic predictors for GC.

## 2. Patient and Methods

### 2.1. Patient

The data of 3085 patients (564 Chinese and 2521 Korean) undergoing curative gastrectomy for GC from 2006 to 2010 in two high-volume hospitals in China (West China Hospital, Sichuan University) and Korea (Severance Hospital, Yonsei University Health System) was collected and analyzed, respectively. Eligibility criteria of patients consisted of (1) histologically diagnosed gastric adenocarcinoma, (2) histologically confirmed R0 gastric resection, (3) curative resection with D2 lymphadenectomy, and (4) absence of neoadjuvant chemotherapy or chemoradiation. Patients with distant metastasis including peritoneal dissemination or who had history of other primary tumors or with multiple primary cancers were excluded from the study. Clinical information about nationality, gender, age, tumor location, tumor size, differentiation of tumor, the depth of tumor invasion, lymph node metastasis, TNM staging, lymphatic invasion, vascular invasion, perineural invasion, Borrmann type, and chemotherapy status was obtained and documented. The West China Hospital Research Ethics Committee has approved retrospective analyses of anonymous data from the database. Signed patient informed consent was waived because of the retrospective nature of the analysis.

### 2.2. Histopathological Evaluation

Tissue samples were obtained from all patients during the surgery and were fixed in 10% formalin, made into paraffin sections, and stained with hematoxylin and eosin in sequence. PNI positivity was identified when carcinoma cells were seen to have infiltrated into the perineurium or neural fascicles. The depth of tumor invasion, lymph node involvement, and distant metastasis, staging, and tumor grade were classified according to the 8^th^ Edition of the *AJCC Cancer Staging Manual*. Clinical pathologists identified the histologic type of gastric carcinoma in line with the histological classification for gastric carcinoma by the World Health Organization (WHO) [16].

### 2.3. Treatments

Curative total or subtotal gastrectomy with D2 lymphadenectomy for GC has been performed for all patients according to the Japanese Classification of Gastric Carcinoma [17]. Fluoropyrimidine alone or a fluoropyrimidine/platinum-based regimen was given to the patients who needed chemotherapy treatments after the operation.

### 2.4. Outcomes

Patients underwent follow-ups conducted by telephone calls, letters, or outpatient visits. Survival status at the last follow-up for Korean patients was also based on data registered in the Korean National Cancer Center. The follow-up information was updated in December 2014 for Chinese patients and March 2014 for Korean patients. The overall follow-up rate was 96.23%. OS was calculated from the date of operation until the date of death or the last follow-up. All terminologies were based on the Japanese Classification of Gastric Carcinoma [18].

### 2.5. Statistical Analysis

Statistical analyses were performed using SPSS 19.0 (SPSS Inc., Chicago, IL) software. The chi-squared test, Fisher exact test, or nonparametric test was used to determine the relationships between the status of PNI and other well-known clinicopathological factors. Survival analysis and curves were presented by the Kaplan-Meier analysis and compared by the log-rank test. Whether PNI would work as a prognostic factor along with other predicting parameters was assessed by the multivariate Cox regression analysis. All *P* values were two-sided in tests, and *P* values less than 0.05 were considered to be statistically significant.

## 3. Results

### 3.1. Patient Characteristics

From January 2006 to December 2010, a total of 3085 patients were reviewed; then, 116 patients were excluded due to lost to follow-up. The mean durations of follow-up were 55.53 months in Chinese patients and 47.76 months in Korean patients. Data of 2969 patients who had received curative gastrectomy for gastric cancer were retrospectively analyzed. Of the 2969 patients, 1997 were male and 972 were female with the mean age of 57.36 ± 12.02. According to the 8^th^ edition TNM staging system, there were 1018 (34.3%) patients classified as stage I, 723 (24.4%) as stage II, and 1228 (41.4%) as stage III. The majority of the tumors were located in the lower third of the tumor (58.6%), 16.7% in the upper third, and 24.4% in the middle third, and only 0.3% of the tumors involved the whole stomach. Most of the patients (66.3%) had tumors with poor differentiation, 27.3% with moderate differentiation, and 6.3% with well differentiation. 2011 (67.7%) patients underwent distal gastrectomy, 74 (2.5%) patients underwent proximal gastrectomy, and 884 (29.8%) patients underwent total gastrectomy. Approximately half of the included patients (47.9%) accepted chemotherapy after surgery (Table 1).

### 3.2. Relationship between PNI and Other Clinicopathological Factors

PNI was positive in 35.5% (1055/2969) of patients. The incidence of PNI was significantly higher in Korean patients (*P* < 0.001) and patients over 60 years old (*P* = 0.028). Tumors with larger size, poorer differentiation, more advanced clinical stage, and lymphatic invasion were more easily to be detected as PNI-positive (*P* < 0.001). Tumor location was also closely related to the incidence of PNI (*P* = 0.001). On the contrary, no association was found between gender and PNI positivity (*P* = 0.055) (Table 1).

### 3.3. Prognostic Significance of PNI in Patients Who Underwent Curative Resection

**Figure 1:**
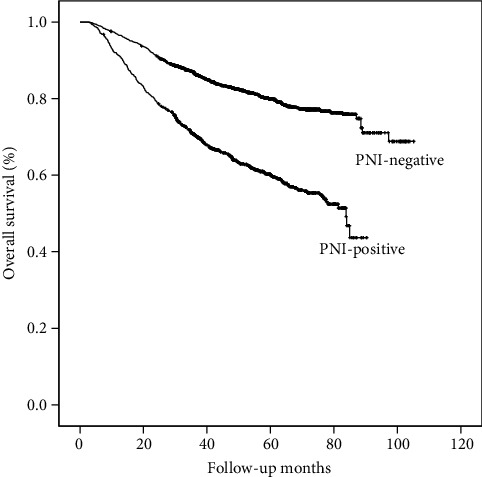
Overall survival curves for patients between the PNI-negative group and the PNI-positive group. The 5-year OS rates were 82.5 and 63.8% in the two groups, respectively (log-rank *P* < 0.001).

**Figure 2:**
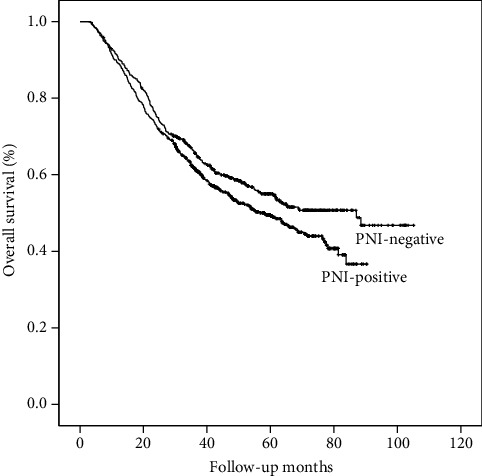
Overall survival curves for PNI-negative and PNI-positive patients with TNM III tumors. The 5-year OS rates were 55.0 and 49.5% in the two groups, respectively (log-rank *P* = 0.032).

Univariate analysis suggested that PNI had impact on the OS of patients with GC undergoing curative resection (Figure 1). The mean survival time of patients with positive PNI (62.5 months) is much shorter than that of patients without PNI (87.3 months) (*P* < 0.001). However, the results of multivariate analysis showed that PNI was not an independent prognostic factor for GC patients. By dividing the patients into subgroups according to the staging and reevaluating the prognostic significance of PNI in patients with different TNM stages, we found that the survival curves showed a significant difference between the PNI-positive group and the PNI-negative group only for patients in stage III (Figure 2), while these survival differences could not be observed in stage I and stage II patients (*P* = 0.48 and 0.69 for stage I and stage II, respectively). Rather than stages I and II, furthermore, positive PNI was proved to be an independent prognostic factor for patients in stage III (*P* = 0.037, hazard ratio: 1.21, 95% confidence interval: 1.01-1.44) in addition to other well-acknowledged clinical-pathological factors including patients' age (*P* < 0.001), pT stage (*P* < 0.001), and pN stage (*P* < 0.001) in the multivariate analysis, while tumor size, tumor differentiation, vascular invasion, and lymphatic invasion were not significant prognostic factors in multivariate analysis (Table 2).

## 4. Discussion

PNI is the neoplastic invasion of perineurium or neural fascicles, and it has been detected in different types of tumors with the incidence ranging from 6.8% to 75.6% in patients [19]. In many malignancies, such as basal cell carcinoma [20], prostate cancer [21], and pancreatic cancer [22], it was an established risk factor related to the OS of patients. It has also been reported that PNI was associated with 1-year recurrence and poor disease-free survival by Fouquet et al. [23]. The prognostic significance of PNI in GC is still controversial, which was discussed in the current study.

A total of 1055 (35.5%) patients' specimens showed positive PNI in this study. Factors including nationality, age, tumor location, tumor size, tumor differentiation, stage, lymphatic invasion, and vascular invasion showed close relationships with PNI in tumors, most of whom have been reported in previous studies [13]. Deng et al. [24] found that PNI was significantly associated with N stage, T stage, and tumor vascular invasion in a systematic review, which was in line with the present analysis. They demonstrated that PNI was irrelevant to sex, age, and tumor location, although the last two factors were considered contrarily in our and some other studies [12, 15, 25, 26]. PNI tended to present positive in the upper third, middle third, and entire stomach in our series, which could be explained by the fact that the proportions of T_3-4_ invasion of tumors located in the upper third (67.5%), middle third (59.4%), and entire (80.0%) stomach were higher than that in the lower third (51.2%) of the stomach. The same phenomenon was documented in the research by Duraker et al. [12]. The influence of patients' age on PNI was not discovered in other studies measuring the prognostic significance of PNI in GC patients. However, Hsieh et al. [25] analyzed the clinicopathological characteristics of gastric carcinoma in 1815 patients and revealed that a significantly higher rate of PNI was found in young (≤40 years) patients compared with older (56-75 years) patients, which corresponded to our analysis. Zhou et al. [26] reported a similar result in their study focused on young Chinese patients. The possible reason might be that the tumors of relatively young patients were often more aggressive and had poorer biological behaviors.

Data from Chinese patients showed that the incidence of PNI (4.5%) was much lower than that in patients from Korea (41.1%) or other previous reports from China [26, 27]. Underreporting by clinical pathologists might be partly responsible for this result. Liebig et al. [28] observed an average of 0.5% of PNI in stage I-IV colorectal cancer in original reports; the detection rate rises to 22% after rereviewing the slides. Peng et al. [29] in their study detected the rate of positivity of PNI in rectal cancer and found that the diagnosis of PNI positivity was missed in 73.8% of patients, compared with the original reports. Fortunately, more attention has been paid to PNI detection for Chinese GC patients currently, and the reported incidence of PNI in pathological examinations has been growing.

Several studies have discussed the prognostic value of PNI in GC, and different opinions were reported in recent years. Tanaka et al. [30] confirmed PNI as a significant prognostic factor in patients with CG, especially for patients with T2 stage. Deng et al. [24] pooled 24 studies in a meta-analysis, which demonstrated that PNI revealed a poor prognosis and affected overall survival and disease-free survival of GC patients who had undergone curative resection. However, significant heterogeneities on the results of overall survival and disease-free survival still exist. Several researchers have also conducted a series of studies that did not recognize PNI as an independent factor predicting outcomes for GC patients [12, 14, 15, 27, 31], and our analysis was in accordance with them. The heterogeneity between studies was probably caused by different ethnicities of patients, types of surgery, degree of lymphadenectomy, staining methods, and interpretation criteria of PNI. We observed that although PNI-positive patients had significantly worse OS than PNI-negative patients in univariate analysis, PNI did not show any additional prognostic value in multivariate Cox regression analysis. It might result from the close relationships between the PNI and advanced T and N stages. Nevertheless, when we divided patients into subgroups and reperformed the Cox regression, PNI was found to play a prognostic role in patients in stage III, along with other factors including age, pT stage, pN stage, and chemotherapy status, indicating that stage III GC patients with positive PNI would have a worse survival outcome than those without. One possible explanation is that PNI was detected much less in TNM I and II stages so that its impact on the OS was veiled during the analysis. Jiang et al. [32] tried to incorporate PNI into the 7^th^ edition TNM staging system. In their study, the difference of survival curves between patients with and without PNI could be found in T4b, N3, and stage III patients, which was consistent with our results. What is notable in the results was that PNI appeared to be a more important prognostic factor than lymphatic and vascular invasion. This finding was somewhat interesting because lymphatic and vascular invasion could be directly related to lymphatic and hematogenous metastasis, while the meaning of perineural invasion was relatively biologically ambiguous. However, similar results were obtained in most of the previous studies [12–14, 33, 34]. On the other hand, some researches that reported lymphatic and vascular invasion as independent prognostic factors along with PNI also failed to include other well-acknowledged factors like age, tumor location, and tumor size in the multivariate analysis, which left the results unlikely to be solid [35, 36]. We considered that the possible reason might be because the prognostic value included in the lymphatic and vascular invasion has been partly compensated by other associated factors, such as the N stage and T stage.

The limitation of this study mainly consisted of its retrospective design, possible deficiency in pathologic examinations, and a lack of data of disease recurrence, which prevented us from further exploring the impact of PNI on disease-free survival of GC patients. Secondly, the regimens and courses of chemotherapy were factors that could influence the prognosis, which were not analyzed. Another limitation was that about >98% of perineural invasion was from the Korean hospital as we have addressed above, which might bias the results. Therefore, we included the institutions as a confounding variable to perform the Cox regression analysis and found that the institutions were not an independent prognostic factor and had no impact on the OS of patients. To our limited knowledge, however, this is the first international multicenter study considering the impact of PNI on GC, and the large sample size could guarantee the relative authenticity of our study.

## 5. Conclusion

PNI occurs frequently in patients with gastric cancer, and the incidence of PNI increases with the staging of tumors. PNI is not an independent prognostic factor for overall GC patients, but the presence of PNI can provide additional information in predicting the survival outcome for those with stage III tumors.

## Figures and Tables

**Table 1:** Association between perineural invasion (PNI) and other clinicopathological factors.

	PNI- (%)	PNI+ (%)	*P* value
Nationality			<0.001
Chinese	428 (22.4)	20 (1.9)	
Korean	1486 (77.6)	1035 (98.1)	
Gender			0.055
Male	1311 (68.5)	686 (65.0)	
Female	603 (31.5)	369 (35.0)	
Age			0.028
<60	1011 (52.8)	602 (57.1)	
≥60	903 (47.2)	453 (42.9)	
Tumor location			<0.001
Upper third	303 (15.8)	192 (18.2)	
Middle third	412 (21.5)	313 (29.7)	
Lower third	1194 (62.4)	545 (51.7)	
Whole stomach	5 (0.3)	5 (0.5)	
Tumor size			<0.001
≤2 cm	531 (27.7)	75 (7.1)	
2.1-5 cm	953 (49.8)	521 (49.4)	
5.1-8 cm	339 (17.7)	334 (31.7)	
>8 cm	91 (4.8)	125 (11.8)	
Differentiation of tumor			<0.001
Well	155 (8.1)	33 (3.1)	
Moderate	576 (30.1)	236 (22.4)	
Poor	1183 (61.8)	786 (74.5)	
pT stage			<0.001
T1	779 (40.7)	18 (1.7)	
T2	405 (21.2)	103 (9.8)	
T3	294 (15.4)	286 (27.1)	
T4a	415 (21.7)	636 (60.3)	
T4b	21 (1.1)	12 (1.1)	
pN stage			<0.001
N0	1084 (56.6)	237 (22.5)	
N1	298 (15.6)	172 (16.4)	
N2	257 (13.4)	229 (21.7)	
N3a	199 (10.4)	262 (24.8)	
N3b	76 (4.0)	154 (14.6)	
Staging			<0.001
Stage I	953 (49.8)	65 (6.1)	
Stage II	457 (23.9)	271 (25.7)	
Stage III	504 (26.3)	719 (68.2)	
Lymphatic invasion			<0.001
Negative	1470 (76.8)	389 (36.9)	
Positive	444 (23.2)	666 (63.1)	
Vascular invasion			<0.001
Negative	1500 (78.4)	371 (35.2)	
Positive	414 (21.6)	684 (64.8)	
Chemotherapy			<0.001
No	1277 (66.7)	269 (25.5)	
Yes	637 (33.3)	786 (74.5)	

**Table 2:** Univariate and multivariate analyses of the prognostic factors in patients with stage III tumors.

Variables	Univariate analysis		Multivariate analysis	
	HR (95% CI)	*P* value	HR (95% CI)	*P* value
Age		<0.001		<0.001
<60	1		1	
≥60	1.39 (1.18-1.63)		1.45 (1.23-1.71)	
Tumor location		0.008		0.047
Upper third	1		1	
Middle third	0.89 (0.70-1.14)		0.87 (0.68-1.11)	
Lower third	0.93 (0.76-1.51)		0.95 (0.77-1.18)	
Whole stomach	3.63 (1.60-8.25)		2.75 (1.18-6.38)	
Tumor size		<0.001		0.18
≤2cm	1		1	
2.1-5cm	0.96 (0.60-1.53)		0.86 (0.53-1.39)	
5.1-8cm	1.39 (0.87-2.22)		1.01 (0.62-1.64)	
>8cm	1.80 (1.11-2.94)		1.10 (0.66-1.84)	
Differentiation of tumor		0.03		0.468
Well	1		1	
Moderate	1.35 (0.66-2.75)		1.18 (0.58-2.43)	
Poor	1.70 (0.85-3.43)		1.32 (0.65-2.67)	
pT stage		<0.001		<0.001
T1	1		1	
T2	0.68 (0.21-2.23)		0.86 (0.25-2.94)	
T3	0.45 (0.14-1.43)		0.74 (0.22-2.45)	
T4a	0.76 (0.25-2.38)		1.27 (0.39-4.15)	
T4b	2.08 (0.63-6.85)		3.43 (0.99-11.91)	
pN stage		<0.001		<0.001
N0	1		1	
N1	0.63 (0.87-4.60)		1.25 (0.17-9.51)	
N2	0.93 (1.30-6.65)		2.21 (0.30-16.54)	
N3a	1.58 (0.22-11.32)		3.59 (0.48-26.78)	
N3b	2.94 (0.41-21.00)		6.60 (0.88-49.31)	
Vascular invasion	1.16 (0.98-1.37)	0.08		
Lymphatic invasion	1.17 (0.99-1.38)	0.07		
Chemotherapy		0.001		<0.001
No	1		1	
Yes	0.73 (0.61-0.88)		0.67 (0.55-0.81)	
Perineural invasion		0.032		0.037
Negative	1		1	
Positive	1.20 (1.02-1.42)		1.21 (1.01-1.44)	
Country		0.38		
Korea	1			
China	1.09 (0.90-1.33)			

## Data Availability

The data that support the findings of this study are available from the corresponding authors upon reasonable request.

## Additional Points

*Synopsis*. This study found that the presence of PNI was not an independent prognostic factor for gastric cancer, except for patients in stage III. The importance of this finding is to provide additional information in predicting the survival outcome for stage III gastric cancer patients.
